# Traditional knowledge of invertebrates used for medicine and magical–religious purposes by traditional healers and indigenous populations in the Plateau Department, Republic of Benin

**DOI:** 10.1186/s13002-019-0344-x

**Published:** 2019-12-16

**Authors:** Laura Estelle Yêyinou Loko, Sédami Medegan Fagla, Azize Orobiyi, Bienvenu Glinma, Joelle Toffa, Omédine Koukoui, Luc Djogbenou, Fernand Gbaguidi

**Affiliations:** 1 Laboratory of Applied Entomology, Faculty of Sciences and Technology of Dassa (FAST-Dassa), National University of Sciences, Technologies, Engineering and Mathematics of Abomey (UNSTIM), BP 14 Dassa-Zoumé, Benin; 2 Medicinal and Organic Chemistry Laboratory, Faculty of Health Sciences, 01, BP 188 Cotonou, Benin; 3 Laboratory of Physics and Synthesis Organic Chemistry (LaCOPS), Faculty of Sciences and Techniques (FAST), University of Abomey-Calavi, BP 4521 Cotonou, Benin; 4 Laboratoire de Physiologie Animale de Signalisation Cellulaire et de Pharmacologie, FAST-Dassa, UNSTIM, BP 34 Dassa Zoumé, Benin; 5 Laboratoire des maladies infectueuses à transmission vectorielle, Institut Régional de Santé Publique, University of Abomey-Calavi, BP 384 Ouidah, Benin

**Keywords:** Ailments, Ethnozoology, Entomotherapy, Local knowledge, Yorùbá, Zootherapy

## Abstract

**Background:**

Since ancient times, invertebrates have played an important role in the traditional medicine in many parts of the world. In south-eastern Benin, more specifically in the Plateau Department, invertebrates are widely used in folk medicine. However, studies on their therapeutic use has been neglected and their magical–religious purposes are poorly understood. The present study aims to document traditional knowledge related to the use of invertebrates for medicinal and magical–religious purposes by traditional healers and indigenous people of Plateau Department.

**Methods:**

An ethno-sociological survey was conducted with 145 informants (80 traditional healers, 12 merchants of medicinal animals and 53 households) belonging to six ethnic groups, in 20 villages located in Plateau of Benin. Data were collected through the participatory rural appraisal method involving individual interviews and direct observations with semi-structured questionnaires. The collected data regarding various medicinal and magical–religious uses of invertebrates were analysed through informant consensus factor (ICF), use value (UV) and, fidelity level (FL).

**Results:**

A total of 20 families and 38 species of invertebrates, distributed among 6 taxonomic categories, were found to be used to treat 50 different ailments. Insects occupied 64.7% of the total invertebrates listed. The African earthworm *Eudrilus eugeniae* K. and African giant snail *Achatina achatina* L. had the highest use values. The highest ICF value (1.0) was cited for diseases of the blood or blood-forming organs. A principal component analysis (PCA) revealed the influence of ethnic groups in the diseases treated with invertebrates. The highest FL (100%) was recorded for 12 invertebrate species treating various ailments. Most of invertebrate-based remedies were associated with plant species. The mode of administration was mainly oral and topical. Most of the invertebrate drugs were traditionally collected in nature or imported, mainly from Nigeria. In addition, 7 magical–religious practices are documented.

**Conclusions:**

Our results reveal that several invertebrate species play an important role in healing practices and magical–religious rituals in the Plateau Department. We suggest further studies to confirm the presence of any bioactive compounds on invertebrate species use in traditional medicine. In addition, this study highlights the need for ecological investigations of these species, in order to develop strategies for their conservation and sustainable use.

## Background

Folk medicine is the source of primary health care for millions of people throughout the world. Although traditional medicine is generally based on the use of plants and plant-derived materials, animals constitute an integral part of the folk pharmacopoeia use in various cultures [1]. Indeed, zootherapy, which refers to the use of animals to treat ailments, and their application for magic rituals and religious practices, involves domesticated and wild fauna resources [2]. According to Marques [3], only 8.7% of the 252 indispensable chemicals selected by the World Health Organization derive from animals. Therefore, animals appear to be a little exploited source of drugs for modern medicine compared with plants [4].

In different regions of Africa, an important use of animals is observed in traditional medicine [5–8]. Animal-based medicines are elaborated from whole animal or parts of the animal body or from animal-derived products [9]. In the Republic of Benin, animals such as mammals, reptiles, fish and birds are widely used in traditional medicine to treat various illnesses and for mystic purposes [7, 10, 11]. While it is known that invertebrates play mystical and magical roles in the treatment of numerous illnesses in a range of cultures [12, 13], the potential medicinal benefits of invertebrates in traditional medicines despite some studies [14–16] have not received the attention they deserve. It is therefore important to document the use of invertebrate species in traditional medicine and healing practices in the Republic of Benin.

Among the twelve Departments of Benin Republic, Plateau Department is distinctive because it is part of the ancient city of Ile-Ife, now known as Yorubaland inhabited by various ethnic groups, including Yorùbá, who possess a broad knowledge regarding the medicinal properties of wildlife species [17–19]. Indeed, in Yorubic traditional medicine, invertebrates such as arthropods are widely used and play a significant role in healing practices, owing to the large number of chemical compounds they synthesize [20]. However, little attention has been given to ethnozoology in Plateau and the use of invertebrates in folk medicine remains unexplored. Knowing that traditional knowledge of animals used in traditional medicine is transmitted from generation to generation through oral folklore [21], it is important to document this indigenous knowledge, which is under threat of erosion due to modernization.

The exploitation of animals as zootherapeutic resources is one of the economic diversification strategies developed by local populations in Benin [7]. Indeed several animals, including invertebrates, are sold in local markets as medicine. However, several invertebrate groups are threatened with extinction although they are rarely considered in conservation policies [22]. Therefore, it is important to identify the invertebrates sold and the supply sources to devise strategies for their sustainable exploitation in Plateau and in other Departments in the Republic of Benin. This study aims to provide an overview of the use of invertebrates in traditional medicine and magico-religious purposes among traditional healers, merchants of medicinal animals and the people of the Plateau Department in the Republic of Benin.

## Methods

### Study area

**Fig. 1:**
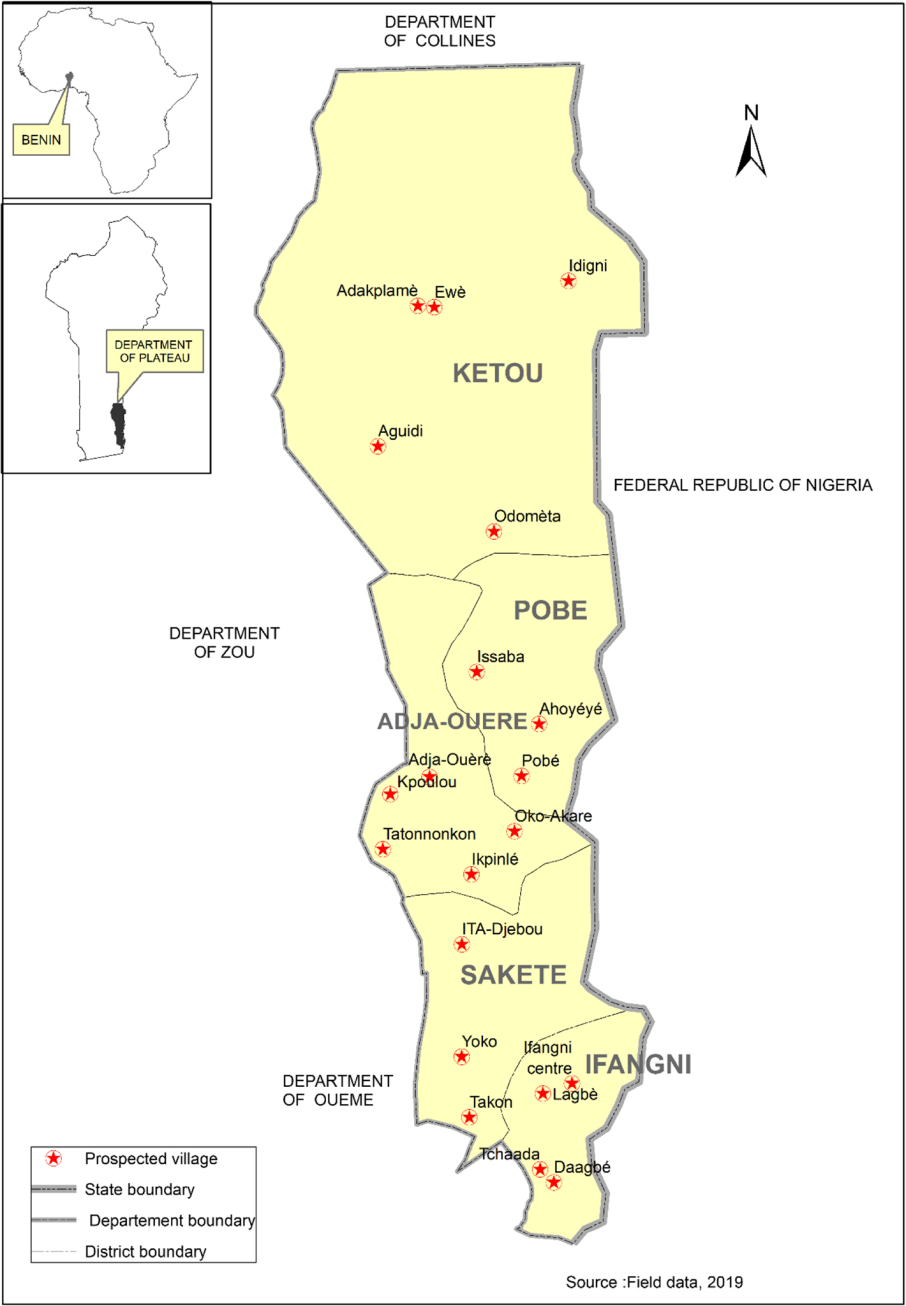
Map of the study area showing the surveyed villages

The Plateau Department (7° 10′ N and 2° 34′ E) is located in south-eastern Benin, bordering Nigeria, and covers an area of 3264 km^2^ (Fig. 1). This Department is subject to an equatorial Guinean coastal climate characterized by four seasons including two rainy alternating with two dry. The average annual rainfall in the area is 1300 mm. The average monthly temperatures are between 25 and 29 °C while the relative humidity of the air oscillates between 68 and 85%. The vegetation is dominated by tree and shrub savannas, shrubby fallows, semi-deciduous forest patches, gallery forests and mangroves. The soil cover consists mainly of ferrallitic red soils formed on the Continental Terminal, vertisols and vertictic soils, hydromorphic soils and tropical ferruginous soils [23]. With a total population of 622,372 inhabitants, the religions practiced in this Department are Christianity, Islam and Animist [24]. Yorùbá-Nago and Holli are the main ethnic groups encountered in the study area. In each of the five municipalities (Kétou, Pobè, Adja-Ouèrè, Sakété and Ifangni) making up the Department, 20 villages were chosen based on two criteria: the ethnic groups they belonged to and accessibility (Fig. 1).

### Survey

Surveys were carried out during the period from December 2018 to March 2019 through the 20 selected villages and public markets of the Department where animals and derivatives are sold for therapeutic purposes. Prior to every interview, we explained the aim of our research and informed consent was obtained from each informant for the use of their knowledge [25, 26]. Data were collected using participatory tools and methods such as individual interview, open discussion and direct observation using a semi-structured questionnaire. Interviews were conducted with the help of local interpreters in the languages of the informants. A total of 145 individuals (80 traditional healers, 12 merchants of medicinal animals and 53 households) belonging to six ethnic groups (Yorùbá-Nago, Goun, Mahi, Tori, Holli, Ouémègbé) were interviewed. The surveyed traditional healers were identified with the help of village chiefs and by using the snowball sampling approach where community members were asked to locate neighbours fitting the criteria [26]. The interviewed traditional healers were only men, ranged in age from 25 to 80 years (average age 56) and the majority (47 people) were illiterate (Table 1). The years of experience in traditional medicine practice of surveyed traditional healers ranged from 4 to 65 years (in average 24 years). The number of surveyed households per village varied from 1–4, with an average size of six individuals per household. Following methodology documented by Mahawar and Jaroli [27], surveyed households were selected based on their recognition as knowledgeable members concerning folk medicine.

**Table 1:** Sociodemographic characteristics of surveyed traditional healers, merchant of medicinal animals and households in Plateau Department

Variables	Interweaved		
	Traditional healers (N* = 80)	Merchant of medicinal animals (*N* = 12)	Households (*N* = 53)
Gender			
Male	80	3	53
Female	-	9	-
Education level			
None	47	7	26
Primary	24	4	19
Secondary	7	1	7
University	2	-	1
Age			
Average	46.6 ± 11.7	49.3 ± 9.2	43.0 ± 9.5
Range	25–80	30–64	26–72
Marital status			
Single	6	7	5
Married	74	5	48
Experience			
Average	23.8 ± 12.7	20.3 ± 7.4	16.5 ± 10.8
Range	4–65	5–30	2–50
Employees			
Average	4.4 ± 5.3	5.0 ± 3.3	-
Range	0–40	0–10	-
Household size			
Average	-	-	6.1 ± 2.3
Range	-	-	1–13
Sociolinguistic groups			
Yorùbá-Nago	57	11	28
Goun	13	-	14
Mahi	3	-	2
Tori	3	-	2
Holli	2	1	4
Ouémègbé	2	-	3

*N number of surveyed people

All surveyed persons in households were men, with age ranged from 26 to 72 years (43 years in average), of whom 26 people were illiterate. The number of years of experience in the use of animals as medicine by surveyed household ranged from 2 to 50, with 16 years on average. Survey data both for traditional healers and households included the sociodemographic characteristics of the interviewees, invertebrates used as remedy (local name, parts used, stage of development used, ailments treated, methods of preparation, administrated singly or in combination with other ingredients, use of live or dead, administered dose), invertebrate storage conditions, collection sites, how knowledge was acquired by the interviewees, any taboo associated with the traditional use of each invertebrates and the use of these animals for magico-religious purposes. In addition, traditional healers were asked to determine which of the invertebrate drugs in their own practices were most commonly prescribed, the most medically valuable and the most expensive [21]. We have translated the ethno-pharmacological uses of each invertebrates into English medical terminologies with the help of educated (university and secondary school level) traditional healers.

Visits were made to the main market of each of five municipalities of the department to interview merchants of medicinal animals. A total of 12 merchants of medicinal animals were interviewed, of whom 9 men and 3 women. The surveyed merchants were mostly illiterate with an average age of 49 years and an average of 20 years of experience in the sale of animals used in traditional medicine (Table 1). The informants were asked to provide the vernacular name, origin, collection sites, conservation mode, commercial value, folk use, parts used and the modes of preparation as well as administration for each invertebrates traded.

Invertebrates revealed by the surveyed individuals were collected and stored in labelled boxes for later identification in the laboratory. With the aid of an insect taxonomist at the Biodiversity Resource Center of International Institute of Tropical Agriculture (IITA-Benin), some insects were identified at the specific level. The remaining zoological materials was identified with the aid of specialists through voucher specimens. Voucher specimens were deposited at the Faculty of Sciences and Technology of Dassa.

### Data analysis

Similarly to Alves et al. [28], the ailments treated by invertebrate remedies were grouped in different categories according to the International Classification of Diseases (ICD-11) used by the World Health Organization (WHO). To determine the extent of utilization of each invertebrate species, we calculated the species use value (UV) following Alves et al. [28] using the formula:

$$ \mathbf{UV}=\sum \boldsymbol{U}/\boldsymbol{N} $$

where *U* is the number of informants mentioning the use of the species and *N* is the number of informants that participated in the survey.

To estimate the level of agreement between interviewees over which invertebrates to use for each category, we calculated the informant consensus factor (ICF) [25]. This factor was calculated according to the formula used by Alves et al. [28]:

$$ \mathbf{ICF}=\frac{\mathbf{nur}-\mathbf{nt}}{\mathbf{nur}-\mathbf{1}} $$

where nur is the number of use reports in each category; nt is the number of species used; and ICF values range from 0 to 1. A high value (close to 1) indicates high consensus, whereby relatively few species are used by many people, and a value near zero indicates a high variation in the use of species for treating a particular illness [25, 28]. ICF was also calculated for each category of ailments, in order to assess the informants agreement on treatments reported for that group of ailments.

To assess the importance of each invertebrate species for the reported ailments, the fidelity level (FL) was calculated following the formula:

$$ \mathbf{FL}\ \left(\%\right)=\frac{\boldsymbol{N}\mathbf{p}}{\boldsymbol{N}}\times \mathbf{100} $$

where *N*p is the number of informants that claim a use of a species to treat a particular disease or ailment and *N* is the total number of informants that use the invertebrates as a medicine to treat any given disease.

The recorded invertebrates were checked against the IUCN Red List Categories (Critically Endangered, Endangered, Vulnerable, Near Threatened, Least Concern, Data Deficient) for assessment of endangered status. Finally, the data obtained were subjected to principal component analysis (PCA) using Minitab 17 software, to describe the relationship between categories of diseases treated with invertebrates and the ethnic groups of the study area.

## Results

### Invertebrates used for medical purposes

**Fig. 2:**
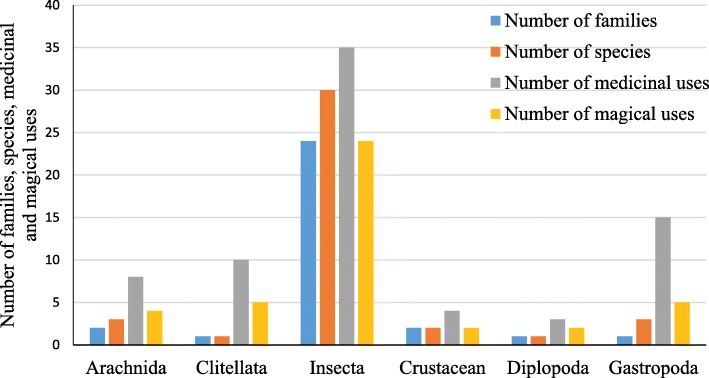
Number of families, species, medicinal and magical uses by taxonomic category

In total, 38 invertebrate species were reported by interviewees as used for 50 medicinal purposes. The reported species were distributed among 20 zoological families. Among them, insects occupied 64.7% of the total invertebrates reported followed by arachnids (8.8%), gastropods (8.8%), clitellata (7.4%), diplopods (7.4%) and crustaceans (2.9%). Insects were the taxonomic group with the largest numbers of animal species and medicinal uses (Fig. 2). The traditional healers prescribed 35 of the 38 invertebrates recorded as used for medicinal purposes, while surveyed households used only 18 of them (Table 2). *Camponotus maculatus* Fabricius, *Phaneroptera nana sparsa* Stål and *Trithemis arteriosa* Burmeister mentioned as home medicinal drug were not used by traditional healers. The great majority of registered invertebrate species (32) have between 1 and 5 different medicinal uses. Only 6 invertebrate species showed more than 5 medicinal uses. The use value of invertebrate species ranged from 0.007 to 0.293 (Table 2). The species which attained the highest use value were *Eudrilus eugeniae* K. (0.293) and *Achatina achatina* L. (0.255). A total of 13 invertebrate species were considered by traditional healers as most prescribed by them (Table 3). Among them, *A. achatina* was mentioned by 29 of the surveyed traditional healers as the most prescribed invertebrate. This gastropod was considered by 7 surveyed traditional healers as having the highest medicinal value.

**Table 2:** Quantitative results for mentioned invertebrate uses in traditional medicine in Plateau Department (*N* = 133)

Species	Family	Local name (ethnic group)	Number of informants mentioning the species			Number of citations			Number of uses	Use value
			TH	H	Total	TH	H	Total		
*Achaea catocaloides* Guenée	Noctuidae	Atotoué (Goun), Awatakpèkpè (Nago)	2	2	4	2	2	4	3	0.030
*Achatina achatina* Linnaeus	Achatinidae	Ogbin (Nago)	22	12	34	24	13	37	13	0.255
*Acraea lycoa* Godart	Nymphalidae	Akpaïkpa (Nago)	2	1	3	3	1	4	3	0.022
*Acraea serena* Fabricius	Nymphalidae	Akpaïkpa (Nago)	2	1	3	2	1	3	2	0.022
*Apate monachus* Fabricius	Bostrichidae	Djegui-Djégui (Nago)	1	-	1	2	-	2	2	0.007
*Apis mellifera* Lineaus	Apidae	Oyin (Nago,Mahi)	6	1	7	10	2	12	6	0.045
*Archachatina marginata* Swainson	Achatinidae	Ogbin (Nago)	6	-	6	6	-	6	5	0.045
*Belonogaster juncea* Fabricius	Vespidae	Ogbon (Nago)	6	-	6	8	-	8	5	0.045
*Brachytrupes membranaceus* Drury	Gryllidae	Irè (Nago)	4	2	6	4	2	6	2	0.045
*Callinectes amnicola* Rochebrune	Portunidae	Alakanran, Agassa (Nago)	6	-	6	7	-	7	5	0.045
*Camponotus maculatus* Fabricius	Formicidae	Lassouga (Holli)	-	1	1	-	1	1	1	0.007
*Ceriagrion glabrum* Burmeister	Coenagrionidae	Agbaroro (Nago)	1	-	1	1	-	1	1	0.007
*Danaus chrysippus alcippus* Cramer	Nymphalidae	Labalaba (Nago)	1	1	2	1	1	2	2	0.015
*Eudrilus eugeniae* Kinberg	Eudrilidae	Ekolo (Nago), Ovoun (Tori, Goun), Vannou-kounongbé (Ouémègbé)	24	15	39	26	15	41	13	0.293
*Formica* spp.	Formicidae	Idjalè, Ekpikpi, Aladi (Nago)	5	-	5	5	-	5	5	0.038
*Hermetia illucens* Linnaeus	Stratiomyidae	Echichi odin (Nago)	1	-	1	1	-	1	1	0.007
*Limicolaria aurora* Jay	Achatinidae	Okoto (Nago)	1	1	2	1	2	3	3	0.015
*Limnephilus* sp.	Limnephilidae	Chèfa-sokpo, Tchègui-sakpo (Nago)	4	-	4	4	-	4	3	0.030
*Lucilia sericata* Meigen	Calliphoridae	Echichi (Nago)	1	-	1	2	-	2	1	0.007
*Luciola discicollis* Castelnau	Lampyridae	Danandanan (Nago)	1	-	1	1	-	1	1	0.007
*Macrotermes bellicosus* Smeathman	Termitidae	Ikan (Nago)	1	-	1	1	-	1	1	0.007
*Mantis religiosa* Linnaeus	Mantidae	Alaguémon (Nago)	1	-	1	1	-	1	1	0.007
*Musca domestica* Linnaeus	Muscidae	Echichi (Nago)	4	3	7	4	3	7	6	0.052
*Myrmeleon formicarius* Linnaeus	Myrmeleontidae	Goulousso (Nago)	2	1	3	2	1	3	2	0.022
*Odontomachus troglodytes* Santschi	Formicidae	Takpèkpè (Nago)	4	-	4	4	-	4	2	0.030
*Oecophylla longinoda* Latreille	Formicidae	Ikarika (Nago), Ahlo (Goun)	4	2	6	4	2	6	5	0.045
*Oryctes monoceros* Olivier	Dynastidae	Kokoro aïtan (Nago), Tran (Goun)	2	-	2	2	-	2	2	0.015
*Pachycondyla tarsata* Fabricius	Formicidae	Iroro, Ororo (Nago)	3	2	5	3	2	5	3	0.038
*Pandinus imperator* Koch	Scorpionidae	Tamitchèkor (Nago)	6	4	10	6	4	10	6	0.075
*Penaeus* spp	Penaeidae	Edé (Nago)	2	-	2	2	-	2	2	0.015
*Periplaneta americana* Linnaeus	Blattidae	Agnan (Nago), Kakaraka (Goun)	6	5	11	8	6	14	6	0.082
*Phaneroptera nana sparsa* Stål	Tettigoniidae	Bossaclé (Ouémègbé)	-	1	1	-	1	1	1	0.007
*Rhynchophorus phoenicis* Fabricius	Curculionidae	Woyiwo (Nago)	2	-	2	3	-	3	3	0.015
*Scarabaeus nitens* Olivier	Scarabaeidae	Agba (Nago)	1	-	1	1	-	1	1	0.007
*Tachypodoiulus niger* Leach	Julidae	Okoukounroun (Nago)	7	-	7	7	-	7	4	0.052
*Trithemis arteriosa* Burmeister	Libellulidae	Agbaroro (Nago)	-	1	1	-	1	1	1	0.007
*Araneus* spp.	Arnaeidae	Elénan-Igbo (Holli, Ouémègbé, Tori)	4	-	4	4	-	4	2	0.030
*Salticus* spp.	Salticidae	Elénan-Ilé (Nago, Holli)	8	-	8	8	-	8	3	0.060

**Table 3:** The most important invertebrate used in traditional medicine in Plateau Department

Species	Most prescribed^a^	Medical importance^b^	Most expensive^c^
*Achatina achatina*	29	7	45
*Apis mellifera*	5	2	1
*Salticus* spp.	4	-	-
*Eudrilus eugeniae*	4	3	-
*Musca domestica*	3	-	-
*Formica* spp.	2	-	-
*Macrotermes bellicosus*	2	-	-
*Belonogaster juncea*	2	-	-
*Achaea catocaloides*	2	1	-
*Myrmeleon formicarius*	1	-	-
*Brachytrupes membranaceus*	1	1	-
*Periplaneta americana*	-	1	-
*Callinectes amnicola*	-	-	2

^a^Number of the 80 surveyed traditional healers in which the invertebrate was the most prescribed

^b^Number of the 80 surveyed traditional healers for which the invertebrate was considered to be the most important medically

^c^Number of the 145 surveyed people (traditional healer, merchant of medicinal animals and households) for which the invertebrate was the most expensive

### Ailments treated with invertebrates

Based on the information obtained from the traditional healers and surveyed households in the study area, all the 50 reported ailments were categorized into 16 categories of diseases (Table 4). The category ‘undefined illnesses’ which includes all diseases with unspecific description of symptoms included the largest number of ailments (15) treated by invertebrates in the study area. Moreover, the majority of recorded invertebrate species (17) is used to treat undefined illnesses (Table 5). Ten (10) invertebrate species are used as remedies for diseases of the skin, nine (09) against diseases of the circulatory system and eight (08) respectively against diseases of the musculoskeletal system, certain infectious and parasitic diseases. Only one invertebrate species is used to treat diseases of the blood or blood-forming organs. Invertebrate-based remedies used for pregnancy, childbirth or the puerperium showed the most important number (34) of use citation after those of undefined illnesses (Table 5). The highest Informant Consensus Factor (ICF) values were for diseases of the blood or blood-forming organs (ICF = 1), pregnancy, childbirth or the puerperium (ICF = 0.88), and for injury, poisoning or certain other consequences of external causes (ICF = 0.87).

**Table 4:** Categories of diseases treated with invertebrate-based remedies in Plateau Department according to the International Classification of Diseases (ICD-11) used by the World Health Organization (WHO) (*N* = 133)

Categories	Diseases and illnesses	Total
Diseases of digestive system	Diarrhoea, ulcer	2
Disease of respiratory system	Asthma, sore throat pain	2
Diseases of nervous system	Epilepsy, paralysis	2
Diseases of the ear or mastoid process	Earache	1
Diseases of the visual system	Myopia	1
Diseases of the skin	Abscess, burn, wound healing, varicella	4
Diseases of the circulatory system	Haemorrhage, haemorrhoids, hypertension, hypotension	4
Diseases of the musculoskeletal system	Arthritis, backache, body aches	3
Diseases of the blood or blood-forming organs	Sickle cell disease	1
Certain infectious and parasitic diseases	Malaria, mumps, leprosy, athlete’s foot, tuberculosis	5
Endocrine, nutritional or metabolic diseases	Diabetes	1
Mental, behavioural or neurodevelopmental disorders	Memory loss, madness	2
Pregnancy, childbirth or the puerperium	Difficult childbirth, fertility	2
Injury, poisoning or certain other consequences of external causes	Snake bite, scorpion sting	2
Conditions related to sexual health	Sexual weakness, fibroma, vaginal infections	3
Undefined illnesses	Alcoholism, tooth decay, tiredness, dizziness, fever, allergy, liver dysfunction, hernia, pains, headache, splenomegaly, stomachaches, swollen feet, enuresis, menstrual cramps	15

**Table 5:** Informant consensus factor categorized by medicinal use of invertebrate-based remedies in Plateau Department (*N* = 133)

Categories	Number of invertebrate species used	Percentage of all species	Use citation	Percentage of all use citation	ICF
Diseases of digestive system	3	9.1	3	1.4	0.50
Disease of respiratory system	2	6.1	3	1.4	0.50
Diseases of nervous system	5	15.2	15	6.8	0.71
Diseases of the ear or mastoid process	2	6.1	3	1.4	0.50
Diseases of the visual system	3	9.1	6	2.7	0.60
Diseases of the skin	10	30.3	17	7.7	0.43
Diseases of the circulatory system	9	27.3	16	7.2	0.46
Diseases of the musculoskeletal system	8	24.2	13	5.9	0.41
Diseases of the blood or blood-forming organs	1	3.0	2	0.9	1.00
Certain infectious and parasitic diseases	8	24.2	10	4.5	0.22
Endocrine, nutritional or metabolic diseases	2	6.1	6	2.7	0.80
Mental, behavioural or neurodevelopmental disorders	6	18.2	8	3.6	0.28
Pregnancy, childbirth or the puerperium	5	15.2	34	15.5	0.88
Injury, poisoning or certain other consequences of external causes	2	6.1	9	4.1	0.87
Conditions related to sexual health	5	15.2	6	2.7	0.20
Undefined illnesses	17	51.5	69	31.4	0.76

**Fig. 3:**
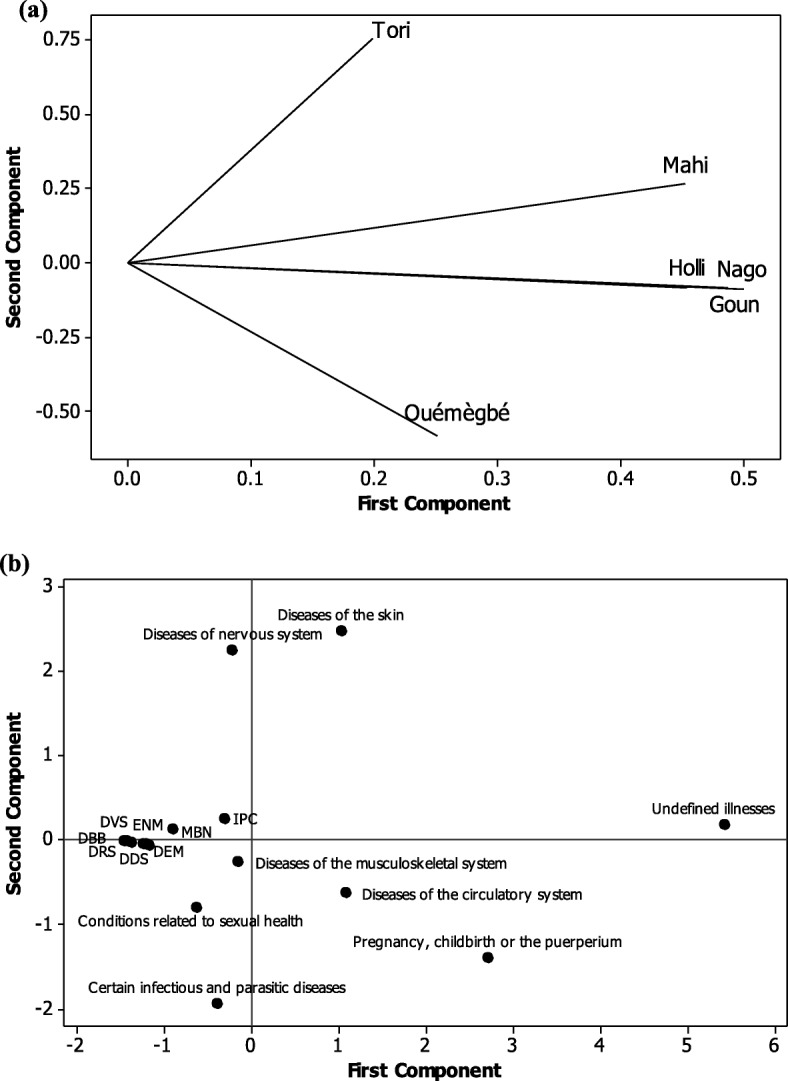
Repartition of diseases treated with invertebrates in the sociolinguistics groups of Plateau Department. **a** Correlation of the plane formed by axes 1 and 2; **b** projection of categories of diseases treated in the plane formed by the axes. DDS, diseases of digestive system; DRS, diseases of respiratory system; DBB, diseases of the blood or blood-forming organs; DVS, diseases of the visual system; ENM, endocrine, nutritional or metabolic diseases; MBN, mental, behavioural or neurodevelopmental disorders; DEM, diseases of the ear or mastoid process; IPC, injury, poisoning or certain other consequences of external causes

Figure 3 shows the results of principal component analysis (PCA) made to determine the relationship between categories of diseases treated with invertebrates and ethnic groups. The results show that the first component explains 56.4% of the information and that the first two components account for 76.5% of the information sought (Fig. 3a). The correlation graph revealed that Mahi, Holli, Yorùbá-Nago and Goun ethnic groups were positively correlated with the first axis (Fig. 3a). The Tori ethnic group was positively correlated with the second axis, and members of the Ouémégbé ethnic group were negatively correlated with the same axis. Our study revealed a difference between ethnic groups of Plateau Department in the use of invertebrates for medicinal purposes (Fig. 3). The projection of the categories of diseases treated with invertebrates in the first two axes shows that Mahi, Holli, Yorùbá-Nago and Goun ethnic groups use invertebrates more to treat undefined illnesses (Fig. 3b), while the Tori ethnic group uses it more for treating diseases of the skin and nervous system. The Ouémègbé ethnic group uses invertebrates more to treat diseases of the circulatory system and for pregnancy, childbirth or the puerperium (Fig. 3b).

### Remedy preparation and administration

Traditional healers have learned to prepare invertebrate remedies through parental heritage (36.2%) or from other traditional healers (63.8%). In surveyed households, indigenous knowledge on invertebrate-based remedies came from parents (67.3%), friends (27.3%) and traditional healers (5.4%). Most of invertebrate remedies were based on the use of the whole animal (91.5%). However, many animal products such as snail without shell (3.6%), snail shell (1.9%), feet (1%), abdomen (1%), snail slime (0.7%) and antenna (0.3%) were used as therapeutic resources as well. Most of invertebrate-based remedies (96.1%) were usually mixed with other drug materials to produce the desired effects. Guinea pepper (15.4% of remedies), palm derivatives (9.8%), moringa leaves (5.7%) and shea butter (4.1%) were the most commonly used plants in combination with invertebrate-based drugs. The results depict 6 modes of preparation for the medicinal invertebrates (Table 6). Powder occupied 47.8% of the total preparations, followed by decoction (36.9%), maceration (6.9%), raw (5.4%), cooked and infusion (each 1.5%). Concerning the forms of administration, the most frequently used mode of remedy administration is oral ingestion (64.2%), followed by topical uses (33.6%), visual (1.5%) and nasal (0.7%) applications. Most invertebrate medicines (67.2%) have no dosage and are taken at will (Table 6).

**Table 6:** List of invertebrates use in traditional medicine in Plateau Department

Species	Ailments	Life stage	Used part	Preparation	Application	Dosage (per day)	Used in combination with	SG	FL
Insecta									
*Achaea catocaloides*	Allergy	Adult	WA	Powder	Topical	3 times	Palm oil	G	50.0
	Headache	Nymph		Powder	Oral	2 spoons	-	N	25.0
	Arthritis	Adult		Decoction	Oral	2 cups	Guinea pepper + moringa fruit	N	25.0
*Acraea lycoa*	Headache	Adult	WA	Powder	Oral, topical	NPD	Shea butter	N	50.0
	Fever			Powder	Topical	NPD	Some plants		25.0
	Diarrhoea			Decoction	Oral	1 cup	Some plants		25.0
*Acraea serena*	Headache	Adult	WA	Powder	Oral	2 spoons	Some plants	N	66.7
	Earache			Raw	Topical	NPD			33.3
*Apate monachus*	Leprosy	Adult	E	Raw	Topical	NPD	Moringa leaves	N	100.0
	Wound healing								100.0
*Apis mellifera*	Dizziness	Adult	WA	Powder	Oral	1 spoon	Some plants	N	50.0
	Tiredness			Powder		1 spoon	Some plants		50.0
	Asthma			Powder		NPD	Some plants		16.6
	Stomachaches			Decoction		1 cup	Guinea pepper		16.6
	Myopia			Powder		1 spoon	Moringa leaves and fruits		16.6
	Madness			Decoction		2 cups	Some plants	M	16.6
*Belonogaster juncea*	Body aches	Adult	WA	Decoction	Oral	NPD	Some plants	N	33.3
	Burn			Powder	Topical	NPD	Shea butter or palm kernel oil	N	33.3
	Mumps			Powder	Topical	NPD	Palm oil	O, N	33.3
	Tiredness			Decoction	Oral	NPD	Some plants	N	16.6
	Arthritis			Decoction	Oral	NPD	Some plants	N	16.6
*Brachytrupes membranaceus*	Snake bite	Adult	WA	Powder	Topical	NPD	Some plants	N	66.6
	Scorpion sting			Decoction	Oral	2 cups			33.4
*Camponotus maculatus*	Alcoholism	Adult	WA	Maceration	Oral	NPD	Local alcohol	H	100.0
*Ceriagrion glabrum*	Stomachaches	Adult	WA	Decoction	Oral	NPD	Some plants	N	100.0
*Danaus chrysippus alcippus*	Fibroma	Adult	WA	Maceration	Oral	2 cups	Pepper + local alcohol	N	50.0
	Headache					2 spoons	Banana + local alcohol		50.0
*Formica* spp.	Ulcer	Adult	WA	Decoction	Oral	NPD	Some plants	N	20.0
	Malaria			Decoction	Oral	NPD	Some plants		20.0
	Jaundice			Powder	Oral	2 spoons	*Aloe vera* + moringa leaves		20.0
	Enuresis			Decoction	Oral	NPD	Some plants		20.0
	Memory loss			Powder	Oral	NPD	Honey + Guinea pepper		20.0
*Hermetia illucens*	External haemorrhoid	Adult	WA	Powder	Topical	NPD	Guinea pepper + palm kernel oil	N	100.0
*Limnephilus* sp.	Memory loss	Adult	WA	Powder	Oral	2 spoons	Honey + Guinea pepper	N	50.0
	Diabetes			Decoction	Oral	2 cups	Guinea pepper + neem leaves		25.0
	Pains			Decoction	Oral	NPD	Some plants		25.0
*Lucilia sericata*	Haemorrhoids	Adult	WA	Powder	Oral, topical	NPD	Guinea pepper + palm kernel oil	N	100.0
*Luciola discicollis*	Myopia	Adult	WA	Powder	Oral	NPD	-	N	100.0
*Macrotermes bellicosus*	Arthritis	Adult	WA	Decoction	Oral	2 cups	Cassia sp. leaves	N	100.0
*Mantis religiosa*	Wound healing	Adult	WA	Powder	Topical	NPD	Some plants	N	100.0
*Musca domestica*	Myopia	Adult	WA	Maceration	Eye drops	NPD	-	N	28.6
	Wound healing			Maceration	Topical	NPD	Some plants	M	14.3
	Haemorrhoids			Powder	Oral, topical	NPD	Shea butter + Guinea pepper	N	14.3
	Madness			Decoction	Topical	NPD	Some plants	N	14.3
	Dizziness			Decoction	Oral	2 cups	Some plants	N	14.3
	Memory loss			Powder	Oral	2 spoons	Some plants	N	14.3
*Myrmeleon formicarius*	Memory loss	Adult	WA	Decoction	Oral	NPD	Indian hemp + Guinea pepper	N	75.0
	Sexual weakness			Decoction	Oral	NPD	Honey + Some plants		25.0
*Odontomachus troglodytes*	Backache	Adult	WA	Decoction	Oral	2 cups	Some plants	N	50.0
	Allergy			Powder	Topical	NPD	Shea butter + Guinea pepper		50.0
*Oecophylla longinoda*	Stomachaches	Adult	WA	Powder	Oral	2 spoons	Some plants	G	33.3
	Backache			Powder	Topical	NPD		N	16.6
	Hypertension			Decoction	Oral	2 cups		M	16.6
	Body aches			Powder	Topical	NPD		N	16.6
	Pains			Powder	Topical	NPD		N	16.6
*Oryctes monoceros*	Backache	Adult	WA	Powder	Topical	NPD	Some plants	N	50.0
	Hernia			Decoction	Oral	2 cups			50.0
*Pachycondyla tarsata*	Sickle cell disease	Adult	WA	Decoction	Oral	NPD	Moringa and baobab leaves	N	40.0
	Earache			Decoction	Ear drops	NPD	Guinea pepper + palm kernel oil		40.0
	Alcoholism			Powder	Oral	1 spoon	-		20.0
*Periplaneta americana*	Alcoholism	Adult	WA	Maceration	Oral	2 cups	Moringa root + Guinea pepper + alcohol	N	45.5
	Fever			Maceration	Oral	2 cups	Some plants	N	18.2
	External haemorrhoid			Powder	Oral	NPD	Some plants	G	18.2
	Internal haemorrhoid			Powder	Oral	NPD	Some plants	G, N	27.3
	Arthritis			Maceration	Oral	NPD	Guinea pepper + alcohol	N	9.1
	Epilepsy			Decoction	Oral	NPD	Some plants	N	9.1
*Phaneroptera nana sparsa*	Athlete’s foot	Adult	WA	Powder	Topical	NPD	Parrot feathers	O	100.0
*Rhynchophorus phoenicis*	Headache	Adult	WA	Decoction	Topical	NPD	Guinea pepper + tomato leaves	N	33.3
	Tooth decay			Powder	Oral	NPD	Honey + quackgrass roots		33.3
	Fever			Powder	Oral	1 spoon	Palm kernel oil		33.3
*Scarabaeus nitens*	Tooth decay	Adult	WA	Decoction	Oral	NPD	Some plants	N	100.0
*Trithemis arteriosa*	Headache	Adult	WA	Decoction	Oral	NPD	Some plants	N	100.0
Gastropoda									
*Achatina achatina*	Fertility	Adult	WA	Decoction	Oral	1 cup	Some plants	G, O, N, H	18.2
	Haemorrhoids		SWS	Powder	Oral, topical	NPD	Palm oil	G, N	12.1
	Wound healing		SS	Powder	Topical	NPD	Shea butter	N, T	12.1
	Difficult childbirth		SWS	Cooked	Oral	NPD	Some plants	N	12.1
	Stomachaches		WA	Powder	Oral	NPD	Alcohol	G, N	9.1
	Myopia		SSL	Raw	Eye drops	NPD	-	G, N	9.1
	Menstrual cramps		WA	Decoction	Oral	1 cup	Some plants	N	6.0
	Paralysis		SWS	Infusion	Oral	2 cups	Some plants + lemon	N	3.0
	Abscess		WA	Raw	Topical	NPD	Some plants + soap	M	3.0
	Haemorrhage		SSL	Raw	Topical	NPD	Some plants	M	3.0
	Varicella		WA	Powder	Rubbed	NPD	Some plants	H	3.0
	Epilepsy		WA	Cooked	Oral	NPD	Some plants	M	3.0
	Swollen feet		WA	Raw	Topical	NPD	Some plants + soap	M	3.0
*Archachatina marginata*	Difficult childbirth	Adult	SWS	Decoction	Oral	NPD	Some plants	N	33.3
	Liver dysfunction		WA	Decoction		1 cup	Some plants		16.6
	Haemorrhoids		WA	Maceration		1 cup	Guinea pepper + some plants		16.6
	Menstrual cramps		WA	Decoction		2 cups	Some plants		16.6
	Splenomegaly		WA	Decoction		2 cups	Guinea pepper + fermented maize + some plants		16.6
*Limicolaria aurora*	Hypertension,	Adult	WA	Powder	Topical	NPD	Tortoise	G	33.3
	Hypotension			Powder	Topical	NPD	Tortoise	G	33.3
	Ulcer			Decoction	Oral	NPD	Some plant	N	33.3
Crustaceans									
*Callinectes amnicola*	Vaginal infections	Adult	WA	Decoction	Oral	2 cups	Guinea pepper + tobacco leaves	N	33.3
	Headache			Decoction		2 spoons	Neem leaves		33.3
	Arthritis			Decoction		2 cups	Bark of caïlcédrat		16.6
	Tuberculosis			Powder		2 spoons	White onion		16.6
	Fever			Decoction		2 spoons	Some plants		16.6
*Penaeus* spp.	Backache	Adult	WA	Powder	Oral	NPD	Some plants	N	50.0
	Tuberculosis			Decoction		NPD	Sugar + chicken egg shell + salt		50.0
Clitellata									
*Eudrilus eugeniae*	Difficult childbirth	All life stages	WA	Powder, decoction, maceration	Oral	NPD	Guinea pepper + alcohol	N, H, G, O, T	45.0
	Epilepsy			Decoction, powder	Oral, topical	NPD	Some plants	N, T, G	12.5
	Diabetes			Decoction	Oral	2 cups	Some plants	N	12.5
	Fever			Powder	Topical	NPD	Palm kernel oil	G	5.0
	Abscess			Powder	Topical	NPD	Some plants	G	5.0
	External haemorrhoid			Powder	Topical	NPD	Some plants	O, M	5.0
	Internal haemorrhoid			Decoction	Oral	NPD	Some plants	O, M	5.0
	Hernia			Powder	Oral	NPD	Some plants	H	2.5
	Sexual weakness			Decoction	Oral	NPD	Some plants	M	2.5
	Stomachaches			Powder	Topical	NPD	Some plants + soap	N	2.5
	Headache			Powder	Topical	NPD	Some plants	N	2.5
	Paralysis			Powder	Topical	NPD	Some plants	N	2.5
	Pains			Powder	Topical	NPD	Palm kernel oil	G	2.5
Diplopoda									
*Tachypodoiulus niger*	Leprosy	All life stages	WA	Decoction	Oral	2 cups	Some plants	N	28.6
	Paralysis		Feet	Powder	Topical	NPD	Palm kernel oil + some plants		28.6
	Alcoholism		WA	Decoction	Oral	1 cup	Tobacco leaves + Guinea pepper		28.6
	Swollen feet		Feet	Powder	Topical	NPD	Some plants		14.3
Arachnida									
*Pandinus imperator*	Snake bite	Adult	WA	Infusion	Oral, topical	NPD	Alcohol + some plants	G, M	30.0
	Scorpion sting			Powder	Topical	NPD	Some plants	M, N	20.0
	Fibroma			Powder	Oral	NPD	Lemon	N	10.0
	Pains			Powder	Oral	NPD	Some plants	H	10.0
	Arthritis		Tail	Maceration	Oral	NPD	Some plants	M, H	20.0
	Abscess		WA	Powder	Topical	NPD	Palm kernel oil	N	10.0
*Araneus* spp.	Headache	Adult	WA	Powder	Oral	NPD	Some plants	O, T, N	75.0
	Arthritis			Powder	Topical	NPD		H	25.0
*Salticus* spp.	Headache	Adult	WA	Powder	Topical	NPD	Some plants	N, H, M	77.8
	Body ache			Powder	Oral	NPD		N	11.1
	Fever			Powder	Oral	NPD		N	11.1

*FL* fidelity level, *SG* sociolinguistic groups, *N* Nago, *G* Goun, *M* Mahi, *T* Tori, *H* Holli, *O* Ouémègbé, *WA* whole animal, *E* excrement, *SWS* snail without shell, *SS* snail shell, *SSL* snail slime, *NPD* no particular dosage

When considering the fidelity level of each invertebrate species for the reported diseases, the most quoted species (FL = 100) were *C. maculatus* (alcoholism treatment), *Macrotermes bellicosus* Smeathman (arthritis treatment), *P. nana sparsa* Stål (athlete’s foot treatment), *Hermetia illucens* L. and *Lucilia sericata* Meigen (haemorrhoid treatment), *T. arteriosa* (headache treatment), *Apate monachus* F. (leprosy and wound healing treatment), *Luciola discicollis* Castelnau (myopia treatment), *Ceriagrion glabrum* Burmeister (stomachache treatment), *Scarabaeus nitens* Olivier (tooth decay treatment) and *Mantis religiosa* L. (wound healing treatment).

### Invertebrates used in magical–religious practices

In the study area, at least one mystical–religious use of invertebrates was noted for all the surveyed ethnic groups, except the Goun ethnic group. A total of 12 magical–religious practices were recorded in the study area (Table 7). Twenty-two invertebrate species are considered by 74 traditional healers and 10 surveyed households as having magical or supernatural properties. Most of the invertebrate species (15) were used by the Yorùbá-Nago ethnic group for protection against evil spirits (Table 7). However, most of the invertebrate species were used for multiple magical purposes. For example, *Apis mellifera* L. was used for protection against evil spirits (FL = 4.3), bewitchment (FL = 11.1), for good luck (FL = 28.6) and use by hunters to reach animals (FL = 100). Similarly, *E. eugeniae* was used for protection against evil spirits (FL = 13.1), bewitchment (FL = 11.1), to have good luck (FL =7.1), and love potions (FL = 50.0). The highest FL value (FL = 100) calculated was for *A. achatina* (used for purification), *Pandinus imperator* Koch (protection against accidents), *S. nitens* (protection against thieves) and *A. mellifera* (use by hunters to reach animals).

**Table 7:** Invertebrates used by traditional healers and households in Plateau Department for magical purposes (*N* = 84)

Roles	Scientific name of invertebrates	Uses	Sociolinguistic groups	Fidelity level
Protection against bad evil spirits	*Achatina achatina*	Make scarification on the skin and pass the powder combined with some plants on	Nago	4.3
	*Apis mellifera*			4.3
	*Callinectes amnicola*			4.3
	*Eudrilus eugeniae*			13.1
	*Musca domestica*			4.3
	*Oecophylla longinoda*			4.3
	*Tachypodoiulus niger*			17.4
	*Salticus* spp*.*			4.3
	*Acraea serena*	Reduce to powder, then mix with palm kernel oil and pass the mixture over the body		4.3
	*Limnephilus* sp.	Drink the decoction mixed with some plants		4.3
	*Macrotermes bellicosus*			4.3
	*Odontomachus troglodytes*			4.3
	*Pandinus imperator*	Reduce to powder and drink with cornmeal		8.7
	*Periplaneta americana*			4.3
	*Aedes aegypti*	Reduce to powder and consume with cooked pumpkin seeds	Mahi, Nago	13.1
Bewitchment	*Achatina achatina*	Make scarification on the skin and pass the powder combined with some plants on	Nago	11.1
	*Apis mellifera*			11.1
	*Tachypodoiulus niger*			11.1
	*Gryllotalpa gryllotalpa*	Drink the decoction mixed with some plants		11.1
	*Eudrilus eugeniae*	Reduce to powder and put the powder on the feet		11.1
	*Salticus* spp.	Reduce to powder and lap up	Holli	22.2
	*Oecophylla longinoda*	Grind and mix with soap to wash	Mahi	22.2
Attract customers or any other person	*Musca domestica*	Reduce to powder and mix with local soap to wash	Mahi, Nago	50.0
	*Acraea serena*		Nago	25.0
	*Phaneroptera nana sparsa*		Nago	12.5
	*Penaeus* spp*.*	Reduce to powder and consume	Nago	12.5
To have good luck	*Apis mellifera*	Reduce to powder and mix with alcohol or honey, then consume	Ouémègbé, Nago	28.6
	*Macrotermes bellicosus*		Nago	14.3
	*Musca domestica*	Reduce to powder and put the powder on 3 scarification made in the chest and behind both hands.	Nago	28.6
	*Aedes aegypti*	Reduce to powder and mix with soap to wash on Monday and Thursday	Ouémègbé	7.1
	*Achatina achatina*	Cook and consume with some plants	Nago	7.1
	*Eudrilus eugeniae*	Reduce to powder and mix with soap to wash	Tori	7.1
To create problems for someone	*Macrotermes bellicosus*	Reduce to powder and mix with Indian hemp and palm oil. Use the mixture in a ritual with incantatory words		33.3
	*Scarabaeus nitens*	Reduce to powder and used with incantatory words	Nago	33.3
	*Belonogaster juncea*	Reduce to powder and mix with soap to wash	Nago	33.3
Purification	*Achatina achatina*	Reduce to powder with some plants and consume	Tori, Nago	100.0
Find a job	*Tachypodoiulus niger*	Reduce to powder with some plants and mix with soap to wash	Nago	33.3
	*Acraea serena*		Holli	33.3
	*Pachycondyla tarsata*	Reduce to powder and put in a perfume. Can also be passed over the eyelids as makeup	Holli	33.3
Love charm	*Eudrilus eugeniae*	Reduce to powder and put in a perfume.	Nago	50.0
	*Musca domestica*			50.0
Make a child speak who has difficulty speaking	*Brachytrupes membranaceus*	Mix in palm oil and consume	Nago	50.0
	*Belonogaster juncea*			50.0
Protection against accidents	*Pandinus imperator*	Mix with some plants and consume	Mahi	100.0
Protection against thieves	*Scarabaeus nitens*	Reduce to powder and put on scarification on the back and chest	Ouémègbé	100.0
Use by hunters to reach animals	*Apis mellifera*	Reduce to powder and put the powder in the barrel of the rifle	Nago	100.0

### Commercialization of invertebrates for zootherapy and ritual practices

The majority of mentioned invertebrate species (31) are not commercialised by merchants of medicinal animals. These invertebrates are directly collected in nature by both traditional healers (76 people) and surveyed households (31). Some traditional healers (53) and surveyed households (22) bought medicinal invertebrates from merchants (Table 8). Invertebrates such as *Brachytrupes membranaceus* Drury, *Tachypodoiulus niger* Leach, *P. imperator*, *M. religiosa* and *A. achatina* were the main medicinal invertebrates traded on market by surveyed medicinal animals merchants. Most of the medicinal invertebrate species (21) commercially sold by medicinal animals merchants are also collected from nature. Nevertheless, certain medicinal invertebrate species such as *A. achatina* and *P. imperator* are reared by 3 medicinal animals merchants and by 13 traditional healers. Some merchants (8) import certain invertebrates such as *B. membranaceus*, *T. niger* and *P. imperator* from Nigeria. It was also observed that some traditional healers (19) buy invertebrates directly in Nigeria. For all surveyed merchants, the sale of medicinal invertebrates is only a small part of their income (less than 10%). However, the great majority of them (11) observed an increase in the demand for medicinal invertebrates compared with the past (5 years ago).

**Table 8:** Source of invertebrates used in traditional medicine of Plateau Department

Source	Traditional healers (N* = 80)	Merchant of medicinal animals (*N* = 12)	Households (*N* = 53)	All informants (*N* = 145)	
				Number	Percentage
Nature	76	12	31	119	49.2
Merchant of medicinal animal	53	-	22	75	31
Nigeria	19	8	-	27	11.2
Rearing	13	3	-	16	6.6
Traditional healers	-	-	5	5	2

* N = Number of surveyed people

The majority of invertebrates (4) are sold and stored dried. Three modes of drying invertebrates have been identified in the study area. Sun drying (83.4%) was the main mode used by informants, followed by drying in alcohol and in maize leaves (each 8.3%). Only 16.7% of medicinal invertebrates are turned into powder for sale. Invertebrate storage tools varied from one informant to another. These are paper (46.1%), bottles (30.4%), jars (6.9%), woven basket (4.3%), tin can (4.3%), leaves (3.5%), calabashes (2.6%) and aluminium cooking pots and clay pots (each 0.9%). According to the majority of merchants (8), dried invertebrates have a long shelf life.

### Constraints of used of invertebrates in zootherapy and ritual practices

Six constraints related to the use of invertebrates in zootherapy and ritual practices have been recorded in the study area (Table 9). The scarcity of invertebrates during dry season was the main constraint for traditional healers (40.4% of responses) and surveyed households (46.2% of responses), while very low demand of certain invertebrates was the most important constraint for medicinal animals merchants (81.8% of responses). The difficulty of conservation of invertebrates was the only constraint common to traditional healers (12.8% of responses), medicinal animals merchants (18.2% of responses) and surveyed households (5.1% of responses).

**Table 9:** Constraints related to the procurement and sale of invertebrates used in traditional medicine

Constraints	Traditional healers (N* = 47)	Merchant of medicinal animals (*N* = 11)	Households (*N* = 39)	All informants (*N* = 97)
Rarity of invertebrates during drought	19	-	18	37
No sales market for invertebrates	-	-	11	11
Difficult to find some invertebrates in the markets	15	-	3	18
High cost	7	-	5	12
Difficult conservation	6	2	2	10
Very low demand	-	9	-	9

*N number of surveyed people

### Conservation status of medicinal invertebrates

In total, 31 out of the 38 invertebrate species encountered were not listed on International Union for Conservation of Nature (IUCN) Red List of Threatened Species. Additionally, six invertebrate species (*A. monachus*, *S. nitens*, *T. arteriosa*, *C. glabrum*, *P. nana sparsa* and *M. religiosa*) were of least concern (LC) according to the IUCN Red List of threatened species, and 1 species, the bee *A. mellifera* was listed as Data Deficient.

## Discussion

The predominance of men among the surveyed healers and households could be explained by the fact that in Yoruba ethnic groups, traditional healers are men and traditional medicine practice is dominated by males due to secrecy in transmitting the knowledge from generation to generation [29–31]. Indeed, to become qualified to practise Yoruba traditional medicine, it would be necessary to go to apprenticeship (ranging from 2 to 30 years) followed by initiation into the Ifa cult [32]. The presence of women among the surveyed merchants of medicinal animals is not surprising because in the Yoruba socio-cultural area, women are more involved in the sale of traditional medicine products [31, 33].

Zootherapy is well established in the Plateau Department where people use invertebrates to treat both common and rare diseases. Our study revealed that 38 medicinal invertebrates were being used in the study area, indicating very rich ethnomedical knowledge of indigenous people of the Plateau Department. Insects were the most important medicinal invertebrates with the most species and uses. In fact, their immunological, antiviral, analgesic, antibacterial, anti-cancer, diuretic, anaesthetic, antioxidant, anti-inflammatory, anti-rheumatic and immunomodulatory properties are well recognised [16, 34, 35]. However, the African earthworm *E. eugeniae* and giant land snail *A. achatina* had presented the highest use value. In fact, like in our study, many peoples throughout the world had use earthworms to treat diseases such as haemorrhoids, arthritis, postpartum weakness, digestive ulcer, earache and epilepsy [36–39]. Earthworms possess antipyretic, antispasmodic, diuretic, detoxifying, antiasthmatic, spermatocidal, antihypertensive and antiallergenic effects [13, 16, 36]. Similarly, *A. achatina* which is known to have hemagglutination potential [40] is also used by Nigerian people to treat haemorrhage, suppression of convulsion and eye problems [41]. Sodjinou et al. [42] also reported a similar use of *A. achatina* by inhabitants of southern Benin for wound healing, to treat epilepsy and difficult childbirth. The widespread use of invertebrates throughout the world suggest that traditional knowledge on zootherapy is to be studied more seriously, in order to lead to the discovery of new sources of drugs [43].

Our study suggests a wide knowledge of the use of invertebrates in medicine in the surveyed households, compared with the number of common medicinal invertebrates used by traditional healers (15 of the 38 invertebrates). Indeed, for most of the surveyed households, knowledge on invertebrate-based remedies comes mainly from forefathers through informal training or verbal discussion. It is known that elderly persons and traditional healers are the custodians of indigenous knowledge systems [44]. In the current context of erosion of the traditional knowledge system, it is important to preserve this medicinal indigenous knowledge for the benefit of future generations. Of note, three of the identified invertebrate species (*C. maculatus*, *P. nana sparsa* and *T. arteriosa*), only recorded in surveyed households, are not known in the literature as medicinal animals. Knowing that the endogenous knowledges of the populations is built on their observations that have stood the test of time [45], it is important to conduct further studies to confirm the presence of any bioactive compounds in traditional remedies based on these three insects.

Our results showed that some invertebrates had high fidelity level (100%), which indicates that all of the use reports mentioned the same method for using the animal for treatment for the same diseases [46]. However, most medicinal invertebrate species (80%) are used by traditional healers and households to treat more than one ailment. This trend is a common practice observed in folk medicine in different parts of the world [46–49], and biological reasons to explain this and the fact that often different invertebrate species are used to treat seemingly identical ailments are given by Meyer-Rochow in [16]. On the other hand, different invertebrate species were used to treat the same ailment. For instance, in our study, ten invertebrate species were used to treat headache and haemorrhoids respectively. The use of different invertebrate-based remedies for the same ailment allows for adaptation to the availability of the possible animals and suggests that these animals can share similar medicinal properties [50, 51]. For instance, skin diseases have been treated with more invertebrates compared with other disease categories. Similarly, invertebrates were more used to treat skin diseases in contemporary Spanish ethnoveterinary medicine [52].

In the study area, ailments included in the undefined illness category were the most treated with invertebrate-based remedies. These results are in accordance with those of Chakravorty et al. [53] who observed that common ailments encountered in day-to-day life were most treated with animal-derived treatments. Diseases of the blood or blood-forming organs have presented the most important ICF. In general, high ICF value allows to identify interesting species for the search of bioactive compounds [54]. However, in this case, the high ICF value reflects the fact that only one species was listed for the treatment of one disease (sickle cell disease), with two use citations, rather than a high cultural importance. The same trend was observed for the category ‘ophthalmological diseases’ where only one disease was treated by one animal with two use citations [47].

Our results also reveal that the categories of diseases treated with invertebrates were influenced by ethnic groups. The main ethnic groups (Yorùbá-Nago, Mahi, Holli and Goun) in the study area mostly used invertebrate-based remedies to treat various undefined illnesses. Similar trends were observed in Brazil, where the community of Queimada city uses more animal-based remedies to treat diseases with unspecific symptoms [20]. The reason why the Tori ethnic group uses invertebrates more for treating diseases of the skin and nervous system and Ouémègbé ethnic group for treating diseases of the circulatory system and for pregnancy, childbirth or the puerperium could be related to the high prevalence of these disorders in the areas these groups occupy.

Some of the invertebrate species used in folk medicine in the study area are also used in very similar ways by people throughout the world [16]. For example, similarly to inhabitants of the Yoruba tribe of southwestern Nigeria, *A. mellifera* is used to treat madness and *Archachatina marginata* Swainson to treat haemorrhoids [19]. Likewise, *M. religiosa* is used by traditional healers and indigenous people in India to treat ear wounds [49]. Moreover, Lawal and Banjo [19] and Costa-Neto [12] report a use of the housefly *Musca domestica* to treat eye problems. The fact that invertebrate species are being used for the same purpose by several communities might indicate their pharmacological effectiveness. However, the treatment of some diseases by invertebrate-based remedies followed folk logic. For example, the centipede *T. niger* with their numerous legs, feet and articulated body segments are used for foot problems such as paralysis and swollen foot. Similar trends are observed in Korean traditional medicine, where centipedes (*Scolopendra* spp.) are used for leg, foot and joint problems [4, 16]. Likewise, the firefly *Luciola discicollis* Castelnau, which possesses a bioluminescent abdomen, is used in the study area to treat myopia. Another reasoning of folk logic in traditional medicine is based on the negative interactions that the invertebrates have with people. For instance, scorpions (*P. imperator*) whose sting causes pain has its venom used to treat pain and snake bites as also observed in Korean traditional medicine [4, 16]. Similarly, in our study, the ant species *Pachycondyla tarsata* Fabricius, which is also called ‘cadaver ant’ because of it’s strong, putrid and smell, is used to treat alcoholism.

The majority of invertebrate-based remedies preparations were used in combination with plant species or plant derivatives for the treatment of single ailment. Similar combinations were also reported by Chakravorty et al. [53], which observed that in members of the Nyishi and Galo tribes (India), the use of treatments solely based on animals or animal products is rare and that treatments involving animal material frequently contain a plant component as well. Some medicinal preparations where both plants and animals are utilized in combination are also reported from Brazil [50] and India [49]. In the study area, the Guinea pepper (*Xylopia aethiopica* (Dunal) A. Rich.) was the most cited plant used in combination with the invertebrate-based remedies. This plant was found to possess anti-microbial [55], anti-fungal [56], anti-helmintic [57], anti-cancer [58], anti-anaphylactic and anti-inflammatory [59], cardiovascular and diuretic [60] activities. The analgesic effect of the fruits of Guinea pepper could explain their mixing with invertebrate-based remedies in the study area to treat pain disorders including arthritis, stomachaches, earaches, headaches, menstrual cramps, haemorrhoids and neuralgia [61]. Oral and topical applications were the most commonly used routes of invertebrate-based remedies application. This finding is in agreement with the result of various zootherapy studies conducted in Brazil [48], India [49] and in fact throughout the world [16].

As in Yoruba medicine of Nigeria, in the study area, health and religion are tightly interrelated [29]. The use of invertebrates for magical–religious practices has been also observed in several other countries throughout the world such as Brazil [2, 20, 24], Nigeria [19], India [62] and Mexico [63]. In our study, invertebrates were in the majority used by the Yorùbá-Nago ethnic group for spiritual protection against evil spirits. Indeed, Yorùbá people believe that illness can be caused by entities such as witchcraft, sorcery, a god or ancestors [64]. Unlike of the Tribal Adi of North-East India, which believed that some insects are representatives of ghosts and evil spirits [29], the surveyed ethnic groups use insects and others invertebrates for their protection. Similarly to this study, the same invertebrate can have several magical uses and one of these species, *M. domestica*, is used by Yoruba tribe of southwestern Nigeria for spiritual protection [19]. The highest FL (100%) presented by four invertebrate species used for magical–religious purposes might give some useful leads for further esoteric research.

The low demand of medicinal invertebrates, which is the main constraint on their sale according to merchants, is not surprising because, like almost everywhere in the world, most of medicinal invertebrates are collected in nature [47]. Except for *M. religiosa*, which is listed as least concern by the International Union for Conservation of Nature (IUCN) Red List of Threatened Species, the other invertebrates sold at the markets of the study area were not listed. Indeed, invertebrates are rarely considered in conservation policies although several species are in the process of extinction [21]. As in the case of medicinal mammals [7], Nigeria is the main importing country of medicinal invertebrates used in the study area. Further studies must be done to evaluate the abundance and distribution of the sold invertebrate species in Benin and Nigeria for a development of conservation strategies. Moreover, in view of the scarcity of some of these invertebrates during the dry season, it is important to develop techniques for rearing the main invertebrates used as medicinal products in the study area.

## Conclusions

This study is the first one in Benin that documents the use of invertebrates in traditional medicine. The results showed that inhabitants from the Plateau Department use several invertebrate species for healing practices and magical–religious rituals. Since most of the invertebrate species used in the study area are not listed in the IUCN Red List of Threatened Species, we suggest that future studies be conducted for their conservation and sustainable use. Further studies must also be done to confirm the presence of any bioactive compounds in invertebrate species used in traditional medicine in the study area.

## Data Availability

Raw and treated data generated during study are available from the corresponding author on reasonable request.
